# The CCCH zinc finger family of soybean (*Glycine max* L.): genome-wide identification, expression, domestication, GWAS and haplotype analysis

**DOI:** 10.1186/s12864-021-07787-9

**Published:** 2021-07-07

**Authors:** Xin Hu, Jianfang Zuo

**Affiliations:** 1 The Key Laboratory for Quality Improvement of Agricultural Products of Zhejiang Province, College of Advanced Agricultural Sciences, Zhejiang A&F University, Linan, Hangzhou, 311300 Zhejiang China; 2 College of Plant Science and Technology, Huazhong Agricultural University, Wuhan, 430070 Hubei China

**Keywords:** Soybean, CCCH zinc finger (zf_CCCH), Expression, Domestication, Haplotype analysis

## Abstract

**Background:**

The CCCH zinc finger (zf_CCCH) is a unique subfamily featured one or more zinc finger motif(s) comprising of three Cys and one His residues. The zf_CCCH family have been reported involving in various processes of plant development and adaptation.

**Results:**

In this study, the zf_CCCH genes were identified via a genome-wide search and were systematically analyzed. 116 *Gmzf_CCCH*s were obtained and classified into seventeen subfamilies. Gene duplication and expansion analysis showed that tandem and segmental duplications contributed to the expansion of the *Gmzf_CCCH* gene family, and that segmental duplication play the main role. The expression patterns of *Gmzf_CCCH* genes were tissue-specific. Eleven domesticated genes were detected involved in the regulation of seed oil and protein synthesis as well as growth and development of soybean through GWAS and haplotype analysis for *Gmzf_CCCH* genes among the 164 of 302 soybeans resequencing data. Among which, 8 genes play an important role in the synthesis of seed oil or fatty acid, and the frequency of their elite haplotypes changes significantly among wild, landrace and improved cultivars, indicating that they have been strongly selected in the process of soybean domestication.

**Conclusions:**

This study provides a scientific foundation for the comprehensive understanding, future cloning and functional studies of *Gmzf_CCCH* genes in soybean, meanwhile, it was also helpful for the improvement of soybean with high oil content.

**Supplementary Information:**

The online version contains supplementary material available at 10.1186/s12864-021-07787-9.

## Background

Zinc finger (Zf) proteins are a large family in eukaryotes. The Zf motifs in proteins are composed of cysteines and/or histidines, which coordinate with a zinc ion to form local peptide structures to meet specific biological functions demands [1]. Zf proteins are interaction with other macromolecules, such as the metal ion zinc, DNA, RNA, proteins and lipids through their Zf motifs [2]. According to the structure and function, Zf proteins can be divided into at least 14 families, such as ERF, WRKY, DOF and RING-finger families [3–6]. The CCCH is a unique subfamily of Zf proteins, which features one or more Zf motif (s) comprised of three Cys and one His residues [7]. CCCH proteins in plant mainly contain one to six CCCH motifs [1, 7–10]. According to the difference in the number of amino acids between the Cys and His residues in the CCCH motif, the consensus sequence of the CCCH motif can be defined as C-X_4–15_-C-X_4–6_-C-X_3–4_-H (X for any amino acid, C for Cys and H for His) [10]. Most of the Zf subfamilies have been identified as DNA-binding proteins or protein-binding proteins [11], while more and more evidence demonstrates that CCCH zinc finger (zf_CCCH) may be RNA-binding proteins functions in RNA processing [12–14].

It has been reported the zf_CCCH family plays a key role involving in various processes of plant development and adaptation. In *Arabidopsis*, the CCCH gene *AtPEI1* is essential for heart-stage embryo formation in *Arabidopsis* seeds [15]. *AtTZF1* was identified to be involved in sugar signaling [16]. The overexpression of *AtTZF1* results in compact plants with late flowering and higher stress tolerance, positively regulating the abscisic acid (ABA)/sugar responses and negatively regulating the gibberellic acid (GA) responses [16]. The CCCH genes *AtTZF2/3/4/5/6* of *Arabidopsis* were close paralogous genes to *AtTZF1* [17, 18]*.* The expression patterns of *AtTZF2/3* genes were similar to that of *AtTZF1* [17]. Unlike *AtTZF1/2/3*, the expression of *AtTZF4/5/6* is seed-specific [18]. The expression level of *AtTZF4/5/6* decreased during seed imbibition and was up-regulated by ABA and down-regulated by GA, suggesting that *AtTZF4/5/6* played critical roles in ABA-, light- and GA-mediated seed germination responses [18]. It is reported that salt stress-inducible Zf protein 1 (AtSZF1) and AtSZF2 negatively regulate the expression of many salt-responsive genes, thus improving the salt tolerance of *Arabidopsis* [19]. HUA1, a CCCH-type zinc finger protein with six tandem CCCH motifs, has been identified as an RNA-binding protein and specifically regulates floral morphogenesis by binding to AGAMOUS pre-mRNA leading to the indirect determination of organ identity [12, 20]. AtCPSF30 was demonstrated to be a nuclear-localized RNA-binding protein that can bind to calmodulin in *Arabidopsis* [13]. In rice, *OsDOS* (a CCCH gene in rice) was detected involved in the negative regulation of the jasmonic acid (JA) pathway, and its overexpression significantly delayed the leaf senescence [21].

*OsTZF1* is a rice ortholog gene to *AtTZF1* [22]. The expression of *OsTZF1* gene was found to be induced by drought, salt, hydrogen peroxide, as well as abscisic acid (ABA), jasmonic acid (JA) and salicylic acid (SA) [22]. *OsTZF1* regulates the pre-mRNA stability of downstream genes by directly binding to U-rich regions in the 3′-UTR, thus delaying seed germination and leaf senescence while improving the tolerance to drought and salt stress [22]. Recently, it has been reported that a cotton CCCH gene *GhZFP1* regulates salt tolerance and disease resistance through interacting with a dehydration and a pathogenesis-related protein, respectively [23]. In soybean, only *GmZF351*, encoding tandem zf_CCCH proteins, has been reported to be involved in improving seed oil accumulation without decreasing seed size through activating lipid biosynthesis-related genes in both transgenic *Arabidopsis* and soybean plants [24]. Genetic and functional analyses reveal that *GmZF351* is a regulator of seed oil content in cultivated soybean that was selected during the process of domestication [24].

Soybean (*Glycine max* (L.) Merr.), an important food and oil crop, accumulates large amounts of oil and protein in seeds and represents one of the main sources of vegetable oil and protein for human food and/or animal feed [25]. Many studies of zf_CCCH family have been carried out at whole genome level in Arabidopsis, rice, and maize [1, 10], as well as recently in switchgrass [26], chickpea [27] and *Brassica rapa* [28]. These studies show that zf_CCCH proteins play important roles in many aspects of plant growth and development, but their functions in soybean have not yet been reported. The publications of genomic sequences of several wild and domesticated soybeans and resequencing of 302 soybean genotypes, provided an opportunity for genome-wide analyses in efforts to discern the functional and evolutionary history of the zf_CCCH gene family. In this study, soybean zf_CCCH genes were identified via a genome-wide search that was based on the whole genome of Williams82 (Wm82.a1.v1.1) [29] and systematically analyzed with regard to phylogeny structure, conserved domain, conserved motifs, chromosome localization, duplication, synteny, and transcription factor (TF) binding sites. The expression of *Gmzf_CCCH* was investigated in different tissues using public RNA-seq data, and the effects of selection during soybean domestication on these genes were also investigated using the 302 soybean resequencing data (including wild and cultivar). The association between *Gmzf_CCCHs* and macro traits such as seed oil content, seed protein content and plant height, and the haplotype analysis in the 164 soybeans of 302 resequencing accessions were studied to gain insights into the function of *Gmzf_CCCH* in soybean. The results of this study provide a foundation for better understanding, future gene cloning and functional studies of *zf_CCCH* genes in soybean and will promote more efficient and effective breeding for seed oil content.

## Results

### Identification of Gmzf_CCCH genes in soybean

**Fig. 1:**
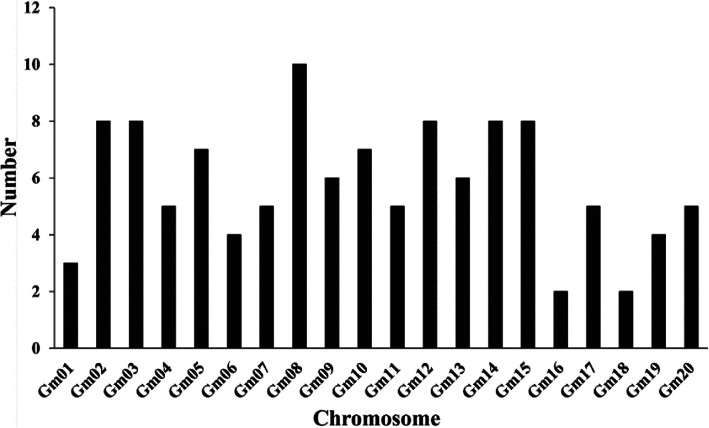
The distribution of the *Gmzf_CCCH* on chromosomes of soybean

In soybean, 116 *Gmzf_CCCHs* were identified via genome-wide search using HMM profiles and confirmed by the conserved domain of CCCH- zinc finger detected in Pfam databases (http://pfam.xfam.org/) and SMART domain search database (http://smart.embl.de/smart/batch.pl). Basic information of all the *Gmzf_CCCH* genes including gene ID, chromosome location, gene length, amino acid length, pI value, MW and subcellular location were also determined and presented in the Table S1. The distribution of the *Gmzf_CCCH* genes on chromosomes were counted and showed in Fig. 1. The number of *Gmzf_CCCH* genes ranges from 2 (Chromosomes Gm16 and Gm18) to 10 (Gm08) on chromosomes (Fig. 1). The Amino acid length of *Gmzf_CCCH* proteins ranged from 127 (*Glyma10g28540.1*) to 1991 (*Glyma14g24710.2*), with isoelectric point and molecular weight ranging from 4.35 (*Glyma13g31033.1*) to 9.88 (*Glyma20g00950.1*) and 14,475.51 Da (*Glyma10g28540.1*) to 220,064.76 Da, respectively (Table S1). The predicted subcellular locations of the *Gmzf_CCCHs* showed that most of the *Gmzf_CCCHs* (104 of 116) were localized in nucleus. Seven *Gmzf_CCCH* genes were predicted to be localized in chloroplast and 3 localized in cytoplasm. The other 2 *Gmzf_CCCH* genes (*Glyma03g02000.3* and *Glyma08g39400.1*) were localized in vacuole and mitochondrial, respectively. Similar result was reported in *Arabidopsis*, Populus and *Brassica rapa*, indicating that most of these proteins might be involved in functions in the nucleus [8, 10]. (Table S1).

### Phylogenetic analysis of Gmzf_CCCH genes

**Fig. 2:**
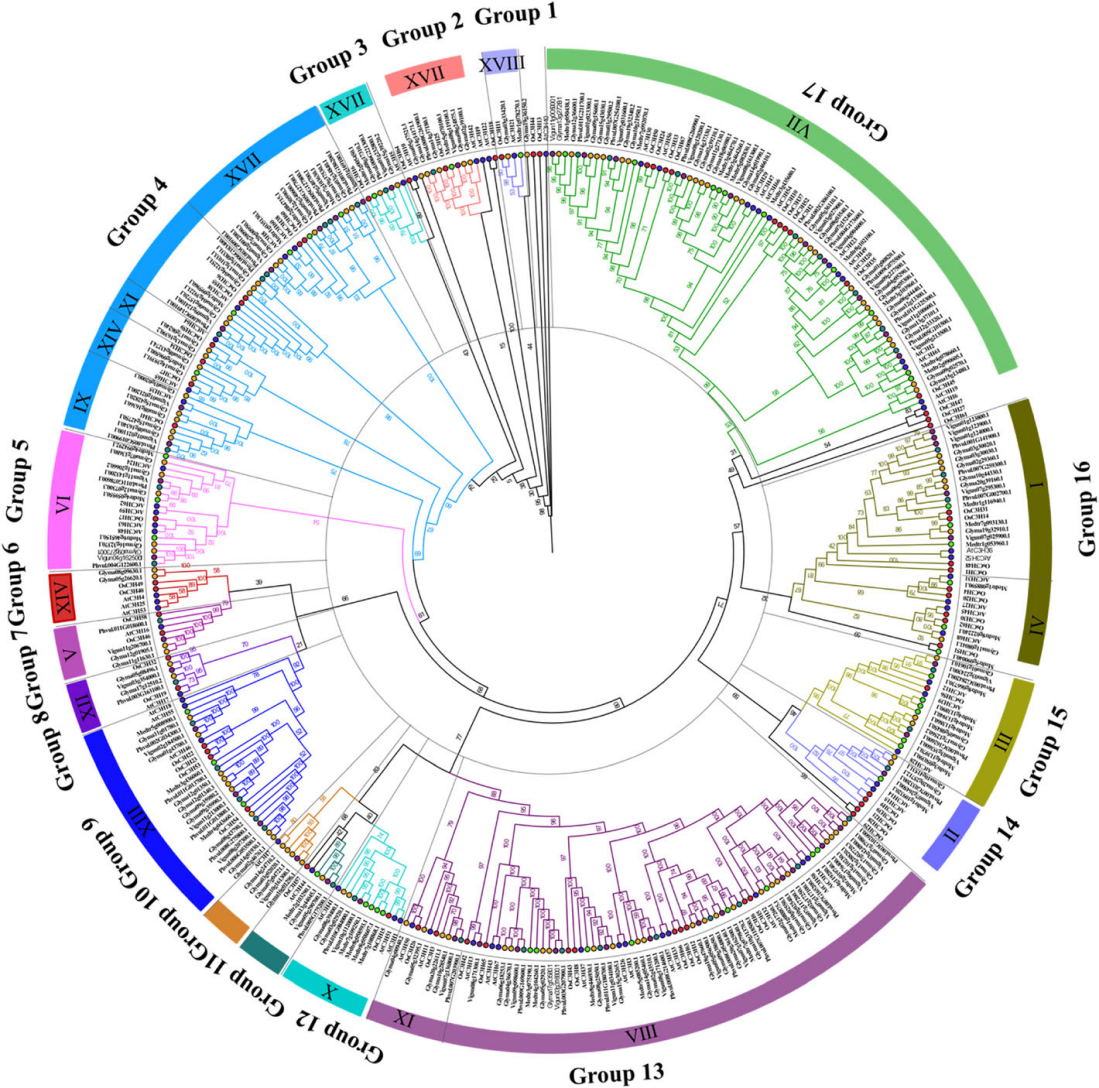
Phylogenetic tree of the zf_CCCH protein sequence of soybean. The full-length protein sequences of 116, 50, 43, 49, 68 and 67 zf_CCCH proteins from soybean, *Medicago truncatula, Phaseolus vulgaris, Vigna unguiculata, Arabidopsis thaliana* and rice respectively, were aligned by MUSCLE and constructed the phylogenetic tree using the maximum-likelihood method in IQTREE with 1000 bootstrap replicates. The numbers beside the branches represent bootstrap values (≥500) based on 1000 replications that were used to class the major 17 subfamilies, subfamily I-XVII in the color circle is based on the reference [10]. The circles at the end of tree nodes marked with blue, red, orange, green, darkgreen and purple colors refer to *Arabidopsis thaliana,* rice, soybean, *Medicago truncatula, Phaseolus vulgaris* and *Vigna unguiculata*, respectively

In order to further investigate the phylogenetic relationships of *zf_CCCH* genes family in soybean and there related legume plants (*Medicago truncatula, Phaseolus vulgaris and Vigna unguiculata*) to other known zf_CCCH gene families (*Arabidopsis thaliana* and rice), a maximum-likelihood phylogenetic tree was constructed using 393 full-length protein sequences of *zf_CCCHs* (116, 50, 43, 49, 68 and 67 for soybean, *Medicago truncatula, Phaseolus vulgaris, Vigna unguiculata, Arabidopsis thaliana* and rice respectively) by IQ-TREE software (Fig. 2). The best-fit model for the construction of the tree was PMB + F + G4. The numbers beside the branches represent bootstrap values (≥500) based on 1000 replications that were used to class the major 17 subfamilies (Group 1–17). According to the consensus classification system (I-XVII) in *Arabidopsis* and rice [10], Group 17 with 26 *Gmzf_CCCH* genes is the largest, which belongs to subfamily VII, followed by Group 13 (belong to VII and IX) with 17 *Gmzf_CCCH* members, and Group 4,10 and 11 with 5,4 and 1 *Gmzf_CCCH* members are new sets, respectively. The distribution of the *Gmzf_CCCH* genes in each group was rather uneven. The specific gene list of each category is presented in the Table S2. Phylogenetic analysis showed that the relationships between legume species and soybean were higher than Arabidopsis and rice.

### Chromosomal locations and Synteny analysis

**Fig. 3:**
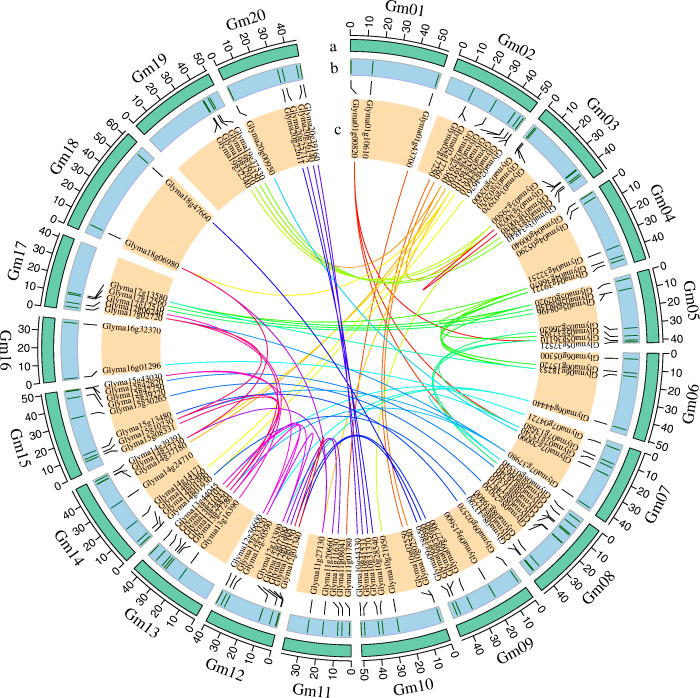
Genomic distribution of *Gmzf_CCCH* genes and gene pairs collinearity analysis in soybean. The tracks toward the center of the circle display: **(a)** Chromosome name and size of soybean (10 Mb tick size); **(b)** The distribution of *Gmzf_CCCH* genes on chromosome; **(c)** Collinearity of *Gmzf_CCCH* genes, tandem and synteny relationships between gene pairs are marked with color lines

Gene duplication, including tandem and segmental duplications, is widespread in plant genomes, which is considered one of the major driving forces of genome evolution resulting in large gene family expansion in plants [30]. Duplicated genes are the source for creating novel genetic variation. MCScanX was used to analyze the gene duplications of *Gmzf_CCCH*s [31]. In total, 88 genes were involved in duplication. Five gene pairs *Glyma02g17250/Glyma02g17260*, *Glyma03g30020/Glyma03g30030*, *Glyma09g35980Glyma09g35990, Glyma10g02550/Glyma10g02540*, and *Glyma12g01340*/*Glyma12g01350*, were identified as tandem duplicated genes and located on chromosomes Gm02, Gm03, Gm09, Gm10 and Gm12 (Table S3, Fig. 3). 75% (87 of 116) of *Gmzf_CCCH*s were involved in segmental duplication forming 68 segmentally duplicated gene pairs (Table S3 and S4). Among which, 30 gene pairs were from 5 groups with 6 gene pairs in each group formed by 4 genes, for example, four genes *Glyma01g00820*, *Glyma05g36110*, *Glyma07g15240* and *Glyma08g03540* formed 6 gene pairs for each other (Table S3, S4 and Fig. 3), and 10 gene pairs were from 4 groups with 2 or 3 pairs in each group formed by 3 genes, for example, three genes *Glyma02g29360*, *Glyma03g30020* and *Glyma19g32910* formed 3 gene pairs for each other. These high collinear genes were clustered in same clade (Fig. 2). Moreover, 28 genes were neither tandem nor segmentally duplicated genes. The above results indicated that the segmental duplication events seemed to play the predominant role in the expansion of the *Gmzf_CCCH*s gene family. Collinear analysis showed that there were several multiplicons (or blocks) of segmented repeated gene pairs between chromosomes, showing the regional collinearity of the chromosomes. For example, chromosomes Gm19 and Gm20 are highly homologous to the long arm of Gm03 and Gm10, respectively, and Gm05 is highly homologous to the short arm of Gm17. Similar results were reported in different studies [29, 32, 33], verifying the duplication and diploidization events in the genome, as well as chromosomal rearrangements which was mentioned in Schmutz, et al. [29].

Three rounds of whole-genome duplication (WGD) events, including γ WGD event about ~ 130 to 240 million years ago (Mya), the legume WGD event about ~ 58 Mya, and the Glycine genus WGD event about ~ 13 Mya, were occurred in the soybean genome evolution, which retained more than 50% of the repeated segments [29, 34, 35]. The general range of Ks value for soybean genome γ WGD events is bigger than 1.5, while that for legume WGD event is 0.3 to 1.5, and that for Glycine genus WGD event is 0 to 0.3 [29, 34, 35]. In total, 68 orthologous gene pairs identified within the pairwise syntenic blocks of MCScanX were used to study the WGD events of *Gmzf_CCCH*s (Fig. 3). We calculated the Ks, Ka and the Ka/Ks ratio between collinear gene pairs (Table S4). The WGD events of most *Gmzf_CCCH*s were at the legume and the Glycine genus WGD events, 57.4% of gene pairs (39 of 68) were divergence during the Glycine genus WGD event, nearly 41.2% of gene pairs (28 of 68) were divergence during the legume WGD event, and 1 gene pair was divergence during the γ WGD event. All 68 synteny pairs with Ka/Ks < 1 underwent purify selection during the soybean genome evolution.

### Gene structure, protein domain, and motif analysis of Gmzf_CCCHs

**Fig. 4:**
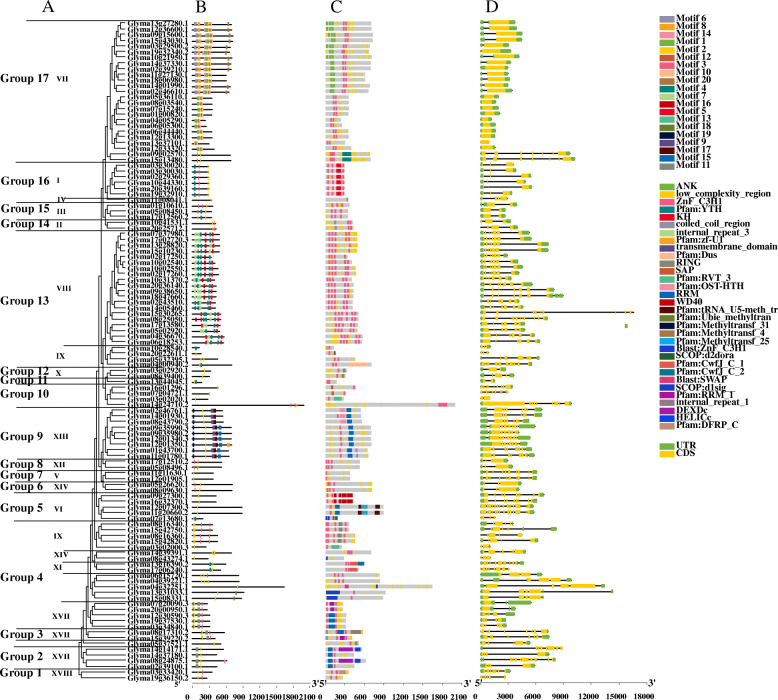
Phylogenetic relationship, conserved motif, protein-conserved domains and gene structure analysis of *Gmzf_CCCH* genes. **(A)** Phylogenetic tree of 116 *Gmzf_CCCH* proteins. The maximum-likelihood phylogenetic tree was constructed using IQ-TREE with 1000 replicates; **(B)** Conserved motifs of *Gmzf_CCCH* proteins. Twenty conserved motifs are shown in different colored boxes, and the details of the motifs are provided in Table S5; **(C)** Conserved domains of *Gmzf_CCCH* proteins, different domain was marked with different colors; **(D)** Exon-intron structures of *Gmzf_CCCH* genes. Orange boxes represent exons, black lines represent introns, and the upstream/downstream regions of *Gmzf_CCCH* genes are represented by green boxes

According to the phylogenetic analysis of *Gmzf_CCCH* genes above and the consensus classification system (I-XVIII) in Arabidopsis and rice [10], the *Gmzf_CCCH* genes of soybean can divided into 13 groups (Table S2, Figs. 2 and 4A). The conserved motifs of the *Gmzf_CCCH* genes were determined by the online MEME suite program (http://meme-suite.org), 20 conserved motifs were detected among the *Gmzf_CCCH* genes varied in length from 11 to 50 aa (Table S5, Fig. 4B). Among which, motif 7,5, 4, 2 and 3 accounting for a large proportion, are CCCH motifs with the constitution sequence of C-X8-C-X5-C-X3-H or C-X5-C-X3-H (Table S5, Fig. 4B). Most *Gmzf_CCCHs* in the same clade shared similar conserved motif composition and showed remarkably similar gene structures (Fig. 4B), suggesting a possible functional similarity. All the detected *Gmzf_CCCH* genes contain the zf_CCCH (PF00642) domains (Fig. 4C). The gene structure of different *Gmzf_CCCHs* including intron-exon size and number varied largely, with the exons ranging from 1 to 14 (Fig. 4D). Closely related members, especially collinear genes, have similar exon-intron structure, and the most important difference among them is the length of intron. For example, the collinear gene pairs *Glyma08g25050.1/Glyma15g30265.1*, and *Glyma13g31033.1/Glyma15g08331.1* are much similar in motif, protein domains and exon-intron structure, while the exon-intron length results in the great difference in gene length (16,596 bp vs 7235 bp, and 14,323 bp vs 6776 bp, respectively) (Fig. 4, Table S1). However, there are great differences in exon/intron and motif composition among different groups, which indicates that functional differentiation exists in each subgroup of soybean *zf_CCCH* gene family.

### Cis-acting elements in the promoters of Gmzf_CCCHs

**Fig. 5:**
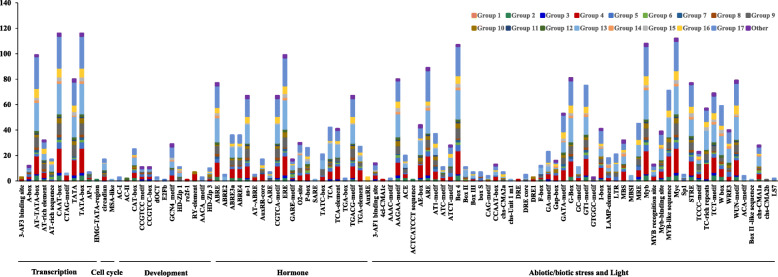
Number of various cis-acting regulatory elements of *Gmzf_CCCH* genes. The cis-acting regulatory elements were identified from PlantCARE online service using 2.0 kb upstream sequence of the transcription start site of *Gmzf_CCCH* genes. The graph was generated using cis-acting element names and functions of *Gmzf_CCCH* genes, different colors refer to the different subfamilies, the ordinate indicates the number of genes containing the cis-acting regulator in different group, the abscissa represents various cis-acting regulatory elements

The cis-acting elements in the promoter regions are the key regions where the transcription factor binding sites initiate transcription and play important roles in the regulation of gene expression. To further explore the possible biological functions of *Gmzf_CCCHs*, the 2 kb upstream promoter regions of all *Gmzf_CCCH*s were submitted to PlantCARE for the prediction of potential cis-acting elements. Virous cis-acting regulatory elements of all *Gmzf_CCCH* genes were predicted to be related to transcription, cell cycle, development, hormones, and stresses (Fig. 5, Table S6). Most *Gmzf_CCCH*s have the cis-acting elements (114 for CAAT-box and 113 for TATA-box) involved in the regulation of transcript. Many cis-acting elements related to hormone signaling pathways were found, such as methyl jasmonate (MeJA), salicylic acid (SA), abscisic acid (ABA), gibberellins (GA) and auxin (IAA). A total of 65 *Gmzf_CCCH*s were detected with MeJA-responsive elements, containing CGTCA-motif and TGACG-motif, and 77 for ABA-responsive element (ABRE), indicating that most of the *Gmzf_CCCH*s might participate in JA- and ABA-mediated signaling pathways. Several cis-acting elements on the promoters of some *Gmzf_CCCHs* are involved in the regulation of tissue-specific expression, such as meristem expression and seed-specific regulation. In addition, some elements were predicted to be involved in various abiotic stresses, such as salt, cold and light, etc.. In particular, all of the *Gmzf_CCCH* genes contained light-responsive elements (Fig. 5, Table S6).

### Tissue-specific expression patterns of Gmzf_CCCHs

**Fig. 6:**
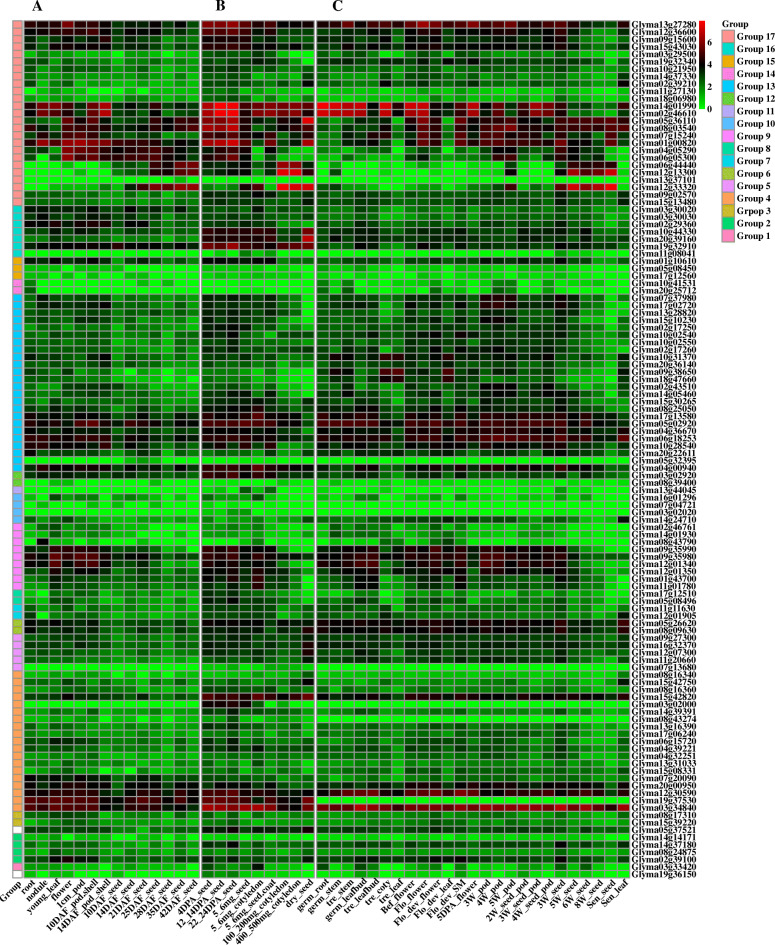
Expression profiles of *Gmzf_CCCH*s in different tissues and organs at different developmental stages. The heatmap was constructed using the ‘pheatmap’ package of R software, and the FPKM (Fragments Per Kilobase per Million) values of *Gmzf_CCCH* genes were transformed by log_2_(x + 1). The red and green colors represent the higher and lower relative abundance of the transcript, respectively. (**A)**, The expression data obtained from Severin, et al. [36], (**B)**: The expression data obtained from Jones and Vodkin [38], (**C)**: The expression data obtained from Shen, et al. [37]

According to the available RNA-seq data of three study [36–38] obtained from Expression Atlas of EMBL-EBI (https://www.ebi.ac.uk/gxa/home), Soybase (https://www.Soybase.org/experiments/) and GEO (Gene Expression Omnibus: https://www.ncbi.nlm.nih.gov/geo/), the temporal and spatial expression patterns of 116 *Gmzf_CCCH* genes in 48 tissues at different developmental stages (including root, nodule, stem, leaf, pod and seed) were visualized using the ‘pheatmap’ package of R (Fig. 6). The expression levels of *Gmzf_CCCH*s varied significantly in different tissues and developmental stages, and the *Gmzf_CCCH*s from the same group showed similar expression patterns. Most of the highly similar genes in sequence, especially collinear (or duplicated) gene pairs in one cluster clade exhibited similar expression patterns. For instance, *Glyma06g44440*, *Glyma12g13300*, *Glyma12g33320* and *Glyma13g37101* were segmentally duplicated genes that are homologous to *ATTZF4/5*(*AtC3H2/AtC3H61*) (Fig. 2), the expression of AtTZF4/5 is reported seed-specific in *Arabidopsis thaliana* [18], similarly, *Glyma06g44440*, *Glyma12g13300* and *Glyma12g33320* were predominantly expressed in seed and very poorly expressed in other tissues in soybean (Fig. 6), suggesting that the three duplicated genes have similar functions in the development of soybean seeds; other four segmentally duplicated genes *Glyma05g36110*, *Glyma08g03540*, *Glyma01g00820* and *Glyma07g15240*, showed similar expression patterns and were mainly expressed at flower, pod and following in seed, which may participate in the regulation of the development of flower, pod and seed. It was worth noting that, several members of *Gmzf_CCCH* family were exhibited expression throughout the reproductive cycle of soybean, and may be involving in regulating the whole growth and development of soybean.

### Selective regions and nearby QTLs around Gmzf_CCCH genes

**Fig. 7:**
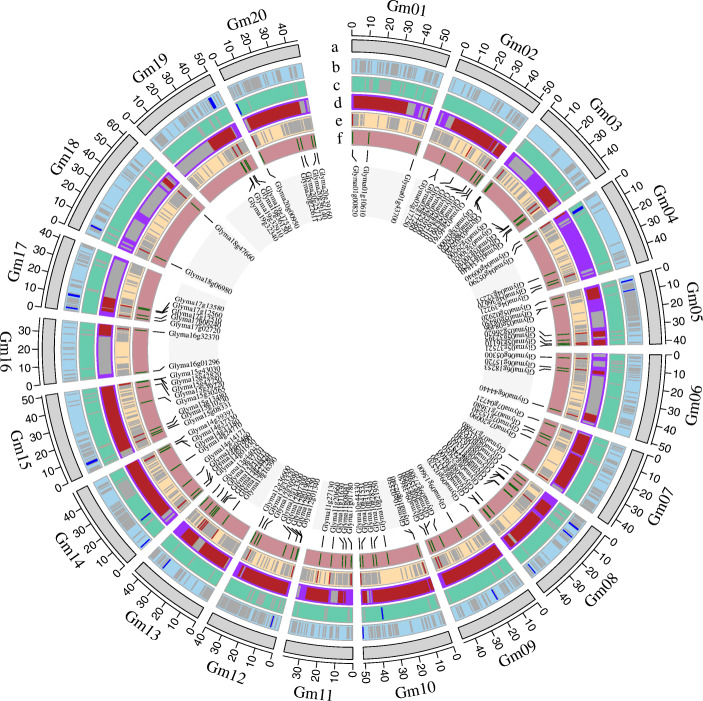
Genomic distribution of *Gmzf_CCCH* genes and previously reported Domestication and improvement regions and QTLs in soybean. The tracks toward the center of the circle display: **(a)**, Chromosome name and size of soybean (10 Mb tick size); **(b),** Previously domestication regions of four studies in soybean (darkgrey lines), the regions around *Gmzf_CCCH* genes were marked with blue; **(c),** Previously improvement regions of four studies in soybean (darkgrey lines), the reagions around *Gmzf_CCCH* genes were marked blue; **(d),** QTL regions form Soybase in soybean (darkgrey lines), the regions around *Gmzf_CCCH* genes were marked with red; **(e),** GWAS regions form Soybase in soybean (darkgrey lines), the regions around *Gmzf_CCCH* genes were marked with red; **(f),** The distribution of *Gmzf_CCCH* genes on chromosome

The soybean domestication and improvement selection regions of four previously reported studies [39–42] were used to analyze whether the *Gmzf_CCCH* genes in the domestication or improvement selection regions. The QTLs of linkage analysis and GWAS were obtained from the soybean database were anchored on the chromosome based on the physic position of associated markers in Wm82.a1.v1.1 genome. The distribution of *Gmzf_CCCH* genes, domestication and improvement selection regions and QTL regions were showed in Fig. 7. Domestication and improvement regions and QTL regions matched with *Gmzf_CCCH* genes were shown in Table S7. Totally, 18 and 4 *Gmzf_CCCH*s were located in the domestication and improvement regions respectively, these genes may have been selected in the process of domestication and improvement of soybean. 84 and 51 *Gmzf_CCCH*s were located in the previously reported QTL regions or around the GWAS loci, and 37 of which were both located in QTL and GWAS regions (Fig. 7, Table S7).

**Fig. 8:**
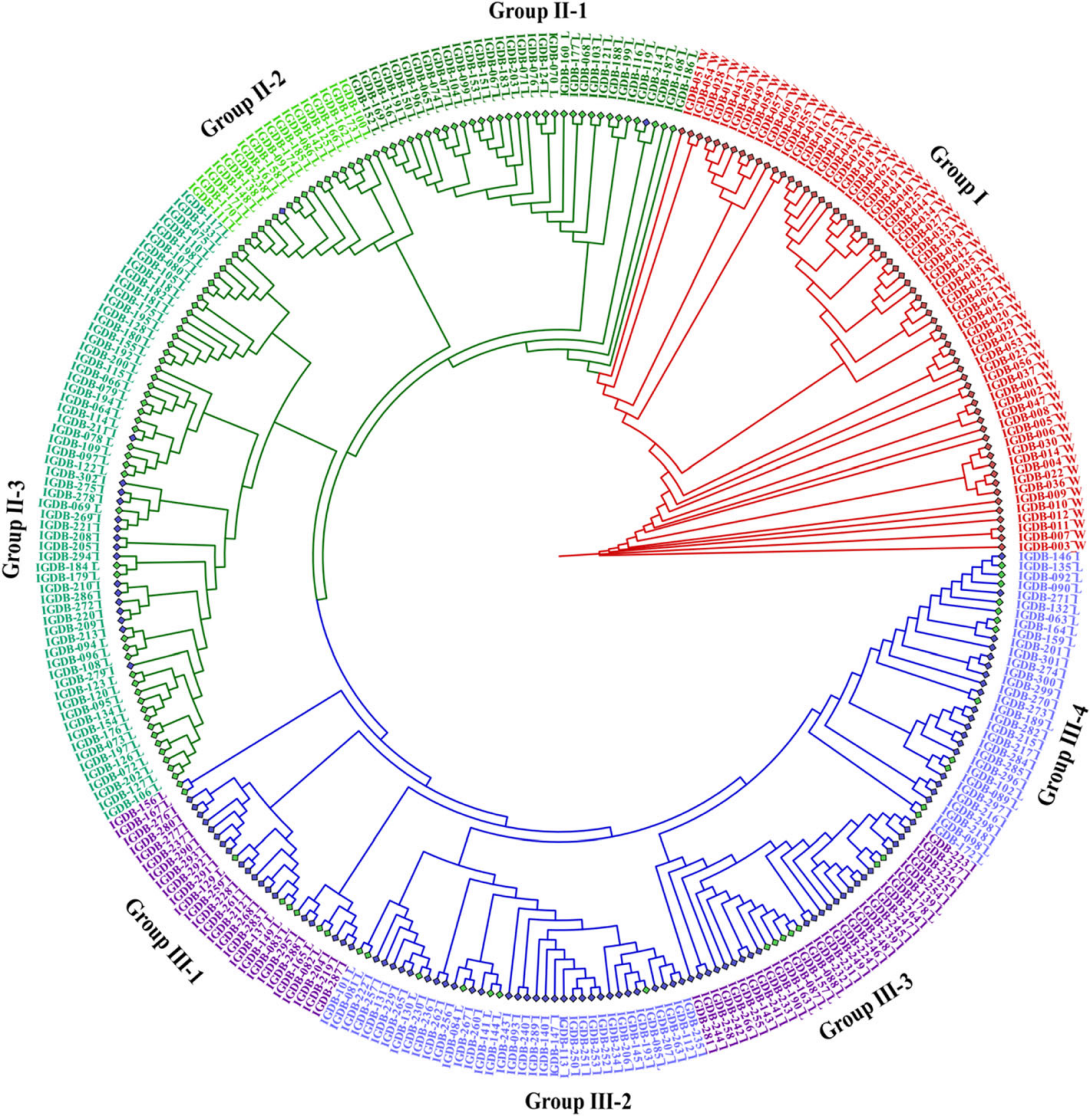
Phylogenetic tree of 302 soybeans. SNPs of the *Gmzf_CCCH* genes among resequenced genomes of the 302 soybean accessions were used to construct the phylogenetic tree using the maximum-likelihood method in IQTREE with 1000 bootstrap replicates. The diamonds at the end of tree nodes marked with red, green and blue refer to wild soybeans, landraces and improved cultivars. Corresponding specie identifiers (IDs) of the accessions are available from Reference [39]

The SNP information of *Gmzf_CCCH* genes obtained from resequencing data of 302 soybeans (Table S8) were used to analyze the clustering and construct the phylogenetic tree (Table S9, Fig. 8). The 302 accessions can be divided into three clusters. Groups I were all wild soybeans (W, 62 accessions were marked with red diamonds), Group II can be divided into three subgroups, including a mixture of most landraces (L, 96 were marked with green diamonds) and 18 improved cultivars (I, marked with blue diamonds), Group III can be divided into four subgroups containing 34 landraces and 92 improved cultivars (Fig. 8). These results imply that the nucleotide diversity of the *Gmzf_CCCH* genes in wild accessions is much higher than that of landraces and cultivated accessions.

### GWAS and haplotype analysis

**Fig. 9:**
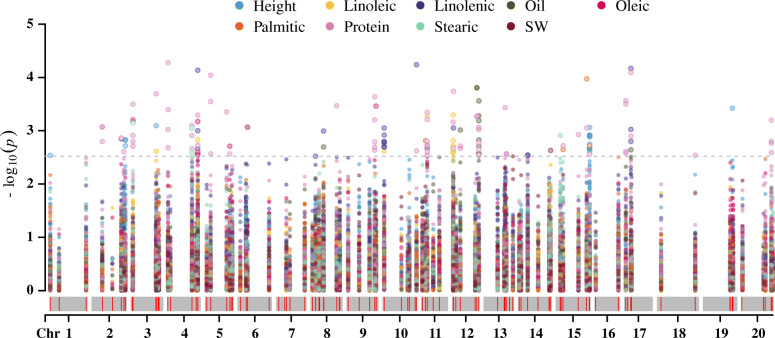
The Manhattan plot of association mapping between 9 traits of soybean and the SNP of *Gmzf_CCCH* genes in 164 of 302 soybean accessions

The GWAS between the phenotypes of 164 soybeans obtained from GRIN and the corresponding genotype data of 116 *Gmzf_CCCH* genes from the resequencing data of 302 soybeans (Including 4176 filtered SNPs on the promoter and gene sequences of *Gmzf_CCCH*) (Table S10, S11 and S12) were carried out by Tassel 5.25 using MLM model. Totally, 162 significant associations were detected between the SNPs loci of *Gmzf_CCCH*s and 9 traits. The associations SNPs for different traits varied largely from 3 for seeds weight to 75 for protein, and the associated *Gmzf_CCCH* genes ranged from 3 to 32 for the 9 traits. If one gene has more than two SNP loci that are significantly associated with a trait, the gene is considered to be a stable associated gene and may be involved in regulating the trait. Therefore, 3, 16, 3, 1, 2 and 7 stably genes were significantly associated with height, protein, oil, oleic, stearic, linoleic and linolenic of soybean, respectively, and may be involved in the corresponding biological processes (Table S13, Fig. 9). The haplotypes of the *Gmzf_CCCH* genes in the 164 soybean accessions were analyzed, and the phenotypic differences corresponding to different haplotypes of the genes were also tested.

#### *The domestication genes of Gmzf_CCCHs detected by GWAS and haplotype analysis*

**Fig. 10:**
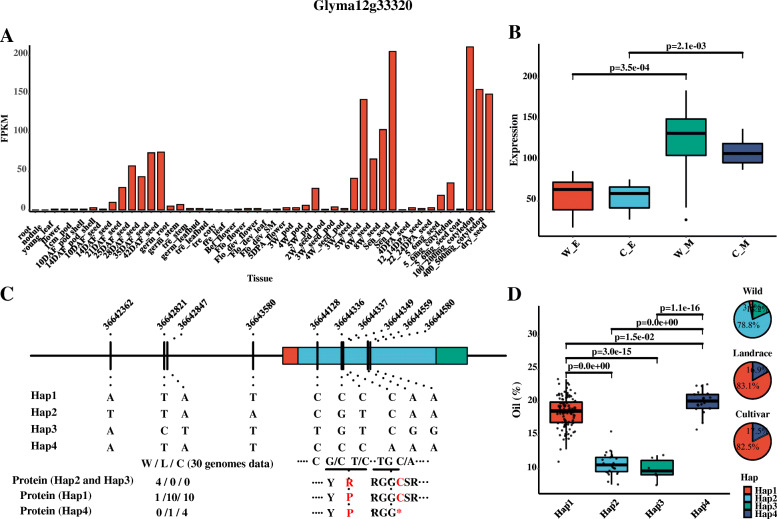
The expression, haplotype, the frequency of haplotype and sequence analysis of *Glyma12g33320*. **(A)**, The expression of *Glyma12g33320* in different tissues and organs at different developmental stages in three studies [36–38]; **(B)**, The expression of *Glyma12g33320* in the early-(E) and mid-(M) maturity during the development of wild (W) and cultivar (C) soybean seed; **(C)**, The sequence analysis of *Glyma12g33320* in the 30 released soybean genomes; **(D)**, The haplotype and its frequency distribution of *Glyma12g33320* among 164 of 302 soybean accessions

Eleven *Gmzf_CCCH* genes were associated with seed oil-related traits, protein, seed weight and plant height of soybean, which had been identified as domestication gene undergone selection during the domestication of soybeans. It was worth noting that *Glyma12g13300*, *Glyma12g33320* and *Glyma12g36600* were detected as domestication genes involving in the regulation of synthesis and metabolism of oil and protein. As an example, for *Glyma12g33320*, previous tissue specific expression analysis showed that *Glyma12g33320* were mainly highly expressed in the late stage of soybean seed development (Figs. 6, 10A), and the study of Lu, et al. [43] proved that this gene showed differentially expression during seed development through gene co-expression networks analysis of 40 transcriptomes from developing soybean seeds in cultivated and wild soybean accessions (Fig. 10B). This indicates that *Glyma12g33320* is involved in the synthesis of oil during seed development. Moreover, The QTL and GWAS data form Soybase showed that *Glyma12g33320* was in the region of previously reported QTLs *mqSeed Oil-018*, *Seed oil 19–2*, *Seed oil 24–28* and *Seed oil 2-g4* (Table S7). Additionally, the haplotype analysis showed that *Glyma12g33320* contained four main haplotypes in 164 soybean accessions, Hap1 and Hap4 were with high oil content, low protein content and low linolenic content (Fig. 10 D, Fig. S1 A1–2), and the percentage of Hap1 and Hap4 were increased from wild (3.0 and 0%) to landrace (83.1 and 16.9%) (Fig. 10D), which further proved that the gene *Glyma12g33320* was involved in the regulation of oil synthesis in soybean and undergone selection in the process of soybean domestication. The sequence analysis of *Glyma12g33320* in the 30 soybean genomes (26 released by Liu, et al. [44] and 4 from soybase) showed that the SNPs at chromosome Gm12: 36,644,336 bp (G to C) and 36,644,337 bp (T to C) resulted in the change of amino acids from arginine (R) in wild (most are Hap2 and Hap3 type) to praline (P) in landraces and improved cultivars (Hap1 and Hap4 type), and the SNPs at Gm12: 36,644,349 bp (C to A) resulted in the early termination of amino acid translation (most are Hap4 type for cultivar) (Fig. 10C and Table S14). Therefore, *Glyma12g33320* was a domestication gene with functional variations in wild, landraces and improved cultivar that affecting the synthesis and metabolism of oil. The soybeans of Hap1 (with amino acids changed) and Hap4 (with amino acids changed and early terminated) are with high oil content, suggesting that *Glyma12g33320* as a zf_CCCH transcription factor may negatively regulated oil synthesis. In addition, this gene is also found to be associated with protein and linolenic, which may be due to the fact that the oil content is continuously improved by positive selection resulting in the inevitably decrease of the protein content, because there is a significant negative correlation between oil content and protein.

*Glyma12g13300*, homologues with *Glyma12g33320*, showed similar seed-specific expression pattern as *Glyma12g33320*, suggesting similar regular pattern in the regulation of oil. The *Glyma12g13300* have four main haplotypes, the accessions with Hap4 showed the high oil content, the percentage of Hap4 were increased from wild (5.6%) to landrace (76.1%), and continual from landrace (76.1%) to improved cultivar (88.2%), while Hap2 and Hap3 was decreased to 0% (Fig. S1 A3–4). The *Glyma12g36600* have five main haplotypes, Hap2, Hap3 and Hap4 were with high oil content and the percentage of these three haplotypes were increased from wild (9.1, 0 and 0%) to landrace (32.1, 41.1 and 25.0%), moreover the percentage of Hap3 was continuously increased from landrace (41.1%) to improved cultivar (95.0%) (Fig. S1 A5–7). This proved that *Glyma12g36600* is related to oil synthesis, and its favorable haplotype was under selection in the process of domestication from wild to landrace soybeans, and the excellent haplotype Hap3 has been further selected and utilized in the process of improvement from landrace to cultivar. *Glyma12g13300* and *Glyma12g3660* have similar functional variations as *Glyma12g33320* at coding region that affecting oil and protein (Table S14). In addition, *Glyma12g13300* and *Glyma12g3660* have variations at promoter region (Table S14), suggesting that they may also affect the synthesis of oil and protein in wild and cultivated soybeans through expression regulation.

The gene *Glyma17g12510* were significantly associated with seed oil and linolenic content in soybean, which was relatively highly expressed in pod formation and early seed development (Fig. 6). It is suggested that the gene is involved in the synthesis of oil and linolenic in the early stage of seed formation. It was worth noting that previous domestication studies indicated that this gene were lying the domestication region of soybean (Fig. 7, Table S7). Haplotype analysis shows that *Glyma17g12510* have seven main haplotypes in the 164 soybean accessions, Hap4 and Hap5 were all wild soybeans with lower oil content but high linolenic content, Hap1 was a major haplotype in landrace and cultivar with high oil content and its frequency increased a lot from wild (7.1%) to cultivated varieties (80% for landrace and 73.5% for cultivar) (Fig. S1 A8–9). These results further verified that the gene *Glyma17g12510* is an oil-related domestication gene, which had been strong positive selected during soybean domestication, resulting in an increased haplotype frequency and oil content of cultivated varieties, and a negative selection for linolenic content at the same time.

*Glyma10g02540* and *Glyma10g02550* was tandem duplicated gene pair significantly associated with linolenic (Table S13, Fig. 7), which showed relatively highly expressed in pod formation and early seed development indicating involving in the synthesis of linolenic (Fig. 6). The haplotype analysis indicated that *Glyma10g02540* and *Glyma10g02550* may also be domestication genes suffering selection during domestication and improvement of soybean (Fig. S1 A10–11), moreover the GWAS data from Soybase indicated that *Glyma10g02540* and *Glyma10g02550* located near to the QTL *Seed oil 5-g3* (about 100 kb) (Table S7, Fig. 7. The tandem duplicated gene pair *Glyma12g01340* and *Glyma12g01350* was associated with linoleic, the homologous gene *Glyma09g35990* was associated with protein (Table S13), These three genes have similar expression pattern during the pod and early seed development (Fig. 6), and their haplotype frequency showed that these genes were under domestication (Fig. S1 A12–14), suggesting that they maybe have similar cross-talk function during seed development.

*Glyma15g42820*, highly expressed in the whole period of soybean growth and development, was significantly associated with height, suggesting that this gene is involved in the growth and development of soybean (Table S13, Figs. 6 and 7). Haplotype analysis showed the gene was under selection during soybean domestication, the frequency of Hap2 were increased in cultivar (Fig. S1 A15). *Glyma13g44045* was associated with seed weight, which was only expressed in the 5–6 mg seed/coat or 3-week seed, indicating that this gene regulated the early development of seeds and ultimately affected the seed weight, and the haplotype frequency showed that the gene was under domestication (Table S13, Figs. 6, 7, FigureS1 A16). Therefore, *Glyma15g42820* and *Glyma13g44045* were domestication genes and involving in regulating the growth and seed development.

#### *The Gmzf_CCCH genes in domestication regions detected by GWAS and haplotype analysis*

Eleven *Gmzf_CCCH* genes were detected significant associated with oil related traits, protein content, seed weight and height, and the haplotype analysis showed that the frequency of haplotypes of the genes had a corresponding change trend from wild to cultivar, suggesting that the genes were under selection during soybean domestication (Table S13, Fig. S1). However, their expressions were no tissue specific (or very low) during the development of seed. This means that these genes may not be directly related to the associated traits, they may be located in the domestication regions near to the key genes for the corresponding traits. Among which, 5 genes, *Glyma05g08450* (associated with protein), *Glyma08g09630* (associated with linolenic), *Glyma08g39400* (associated with linolenic), *Glyma17g02720* (associated with protein) and *Glyma05g02920* (associated with linolenic) were reported in the previous domestication studies of soybean [39–42] (Fig. 7, Table S7), which have been proved located in domestication regions suffering selection by haplotype analysis in this study. For example, *Glyma08g09630* was associated with linolenic content and has four main haplotypes, Hap1 and Hap2 were all wild accessions with high linolenic content and their frequency were increased from wild to cultivar (Fig. S1 B3), suggesting that this gene had been selected. However, this gene showed no tissue specific expression during seed development, which expressed similar in all tissues (Fig. 6). Therefore, this gene might not be directly related to linolenic, but only located in a domestication region near to a key domestication gene that affected the content of linolenic. Similar results were detected for *Glyma05g08450*, *Glyma08g39400, Glyma17g02720, Glyma05g02920* (Fig. 7, Table S7, Fig. S1 B1–5). Moreover, although some *Gmzf_CCCH* genes haven’t been reported in the previous domestication studies [39–42] (Fig. 7, Table S7), the haplotype analysis identified that 6 *Gmzf_CCCH* genes may be under selection at domestication region during soybean domestication. For instance, *Glyma11g27130* was significantly related to linolenic and oil content, which had seven main haplotypes. From wild species to landrace and then to cultivated species, the frequency of haplotype has changed accordingly, the frequency of Hap1 is increasing, which shows that this gene has been strongly selected (Fig. S1 B6–7). similar results were identified for *Glyma03g30030* (associated with protein and height), *Glyma04g32251* (associated with linolenic and protein), *Glyma11g11630* (associated with olic and linolenic acid), and *Glyma12g01905* (associated with protein) and *Glyma19g36150* (associated with height) (Table S13, Fig. S1 B8–15). These genes were not expressed (or very low) during the whole growth period of soybean, implying that these gene may also be located in a domesticated region related to corresponding traits.

## Discussion

The CCCH zinc finger family is a large multifunctional protein family. In plants, *zf_CCCH* genes have been reported to play pivotal roles in cell fate specification and hormone-regulated stress responses. Family members identification and functional studies of the *zf_CCCH* family have been carried out in many model plants and crops [1, 10, 16, 21, 27, 28]. However, the functions of soybean *Gmzf_CCCHs* are rarely reported. In this study, we aim to investigate gene structures, protein properties, phylogenetic relationships, replication characteristics, expression profiles, selection pressures and possible functions of *Gmzf_CCCH* gene family members in soybean. In order to predict and analyze the function of the detected *Gmzf_CCCHs,* many methods and approaches have been used, such as gene expression profiling with multiple transcriptome data, domestication selection, GWAS and haplotype analysis, as well as the gene sequences analysis from pan-genomic data of soybean, A total of 8 *Gmzf_CCCHs* were detected as domestication genes involved in regulation of oil synthesis during the domestication of soybean. Our research provides new insight for future identification and function analysis of this gene family. In addition, the detected oil synthesis-related genes and their excellent haplotypes could be used for improving oil content in soybean breeding.

### Identification and characterization of zf_CCCH gene family help to better understand the evolutionary events and relationships in soybean and its relative species

Typical *zf_CCCH* genes usually have one to six CCCH motifs [1, 7–10]. A total of 116 *Gmzf_CCCH* genes were identified and confirmed by detecting the CCCH- zinc finger domain in the soybean genome of Williams 82, which is higher than those detected in maize (68) [1], rice (67) and *Arabidopsis* (68) [10], chickpea (58) [27]*, Brassica rapa* (63) [28] and switchgrass (103) [26], suggesting the genome size does not determine the number of *zf_CCCH* genes reported for species. However, the number of *zf_CCCH* genes detected in *Medicago truncatula*, *Phaseolus vulgaris* and *Vigna unguiculata* (50, 43 and 49, respectively), was less than a half compared with that in soybean (116). Meanwhile, the genome sizes of *M. truncatula*, *P. vulgaris* and *V. unguiculata* (about 500 Mb, 587 Mb, 620 Mb) are about half to that of soybean (about 1.1 Gb), suggested that the number of *zf_CCCHs* was related to genome size in its relative species. Phylogenetic analysis showed that the relationships between legume species and soybean were closer than *Arabidopsis* and rice. Further, each *zf_CCCH* of the three legume species may find at least one homolog *zf_CCCH* gene in soybean, suggested that soybean experiences an independent Glycine WGD event during the evolution of legumes. This was confirmed by further analyses of genome-wide duplication (GWD), homologous, and historical duplication events of *Gmzf_CCCH* genes. Our results revealed that the *zf_CCCH* genes of soybean are an ancient gene family that expanded as early as ~ 150 Mya ago during the γ WGD event and continued to expand in subsequent legume WGD and Glycine WGD events, and 57.4% of gene pairs (39 of 68) of soybean *zf_CCCH* genes were divergence during the Glycine genus WGD event. Synteny analysis and the physical location of *Gmzf_CCCH* genes show that segmental duplications (75%) were the main mechanisms of *zf_CCCH* gene duplication, which is consistent with the analysis of CCCH families in Arabidopsis, rice and maize [10]. Furthermore, several multiplicons (or blocks) of segmental duplication gene pairs between chromosomes were detected, showing the regional collinearity between chromosomes (such as Gm19 and Gm03, Gm20 and Gm10, as well as Gm05 and Gm17) (Fig. 3), similar results were reported in different studies [29, 32, 33], verifying the duplication and diploidization events in soybean genome evolution, as well as chromosomal rearrangements which was mentioned in Schmutz, et al. [29].

### Several Gmzf_CCCHs detected undergone selection during the domestication of soybeans might be involved in regulation of soybean oil synthesis in different ways

The expression profiles of *Gmzf_CCCHs*, GWAS and haplotype analysis showed that 8 genes (*Glyma12g13300, Glyma12g33320, Glyma12g36600, Glyma17g12510, Glyma10g02540, Glyma10g02550, Glyma12g01340,* and *Glyma12g01350*) were detected as domestication genes involved in regulation of oil synthesis during the domestication of soybeans (Table S7, Fig. S1 A). The genes with different functional mutations in population may result in phenotypic variances through different way. The mutations in the coding region affect the amino acids translation, which lead to gene function change or loss, while the variations of promoter region often affect the expression pattern of genes. For instance, the SNP mutations in the coding region of *Glyma12g13300* resulted in early termination of amino acids translation in different haplotypes, leading to the changes or loss of gene function (Fig. 10, Table S14), thus affecting the oil synthesis and metabolism. In addition, *Glyma12g13300* and *Glyma12g3660* showing high and specific expression in seed (Fig. 6), have variations in both the coding and promoter regions (Table S14), suggested they may affect the synthesis of oil in wild and cultivated soybeans through functional variation and expression regulation. Moreover, In the study of Li, et al. [24], *GmZF351* (*Glyma06g44440*), encoding tandem CCCH zinc finger proteins, was reported as a domestication-selective regulator that was involved in improving seed oil accumulation without decreasing seed size in both transgenic *Arabidopsis* and transgenic soybean plants through the activation of lipid biosynthesis-related genes. Interestingly, the association between *Glyma06g44440* and oil-related traits was not significant in the GWAS of this study, probably owning to population differences. The *Glyma12g13300* and *Glyma12g33320* had been proved to be domesticated genes associated with oil content, which were homologue with *Glyma06g44440*, suggested they may have similar functions or regulation mode in soybean oil accumulation as *Glyma06g44440*. The SNP variations at promoter region or 5′ UTR of *Glyma17g12510*, *Glyma10g02540*, *Glyma10g02550*, *Glyma12g01340* and *Glyma12g01350* may affect the synthesis of oil in the development of seed in wild and cultivated soybeans through expression and transcription regulation (Table S14). Although the function of the detected domesticated genes has been predicted by bioinformatic analyses and supported by multiple data in this study, their specific roles and mode of action need to be investigated by further intensive functional studies, such as the transgenic and biological approaches.

### The co-selection of seed oil and protein content in the domestication of soybean and the utilization of seed oil related elit haplotypes of Gmzf_CCCH genes in soybean breeding

Agronomic traits of soybean have undergone strong artificial selection during the domestication process and modern breeding practices, such as the greatly increased seed oil content from wild to landrace, and further to cultivated soybean [24, 45–47]. In this study, a total of 8 *Gmzf_CCCH* genes were detected as domestication genes involved in regulation of oil synthesis during the domestication of soybeans, among which, *Glyma12g13300*, *Glyma12g33320* and *Glyma12g36600* were significantly associated with oil and protein content, the haplotype analysis showed that the frequency of haplotype with high-oil / low-protein content (Hap1 and Hap4) were increased from wild to cultivated soybean (Fig. 10 and Fig. S1), suggested that co-selection exists between oil and protein content. That could be explained by the following reasons: one is pleiotropism, these genes with higher expression during soybean seed development, are likely to be transcription factor involved in regulating downstream genes and pathways that affecting oil synthesis and protein accumulation; the other is linkage drag, these oil-related genes are located in a strong domestication region (or linkage bloke, LD) where they co-exist other genes controlling protein synthesis, therefore, the protein content is co-selected during the domestication of oil content; another is the correlation between traits, as the main qualitative components of soybean seeds, the content of oil and protein showed significant negative correlation with each other, therefore, during the process of positive domestication of oil from wild to cultivated species, protein content will obviously be subject to certain negative selection, even the protein- and the oil-related genes are not in the same domesticated region. Thus, strong signatures of selection associated with key traits can also cause co-selection for related traits even in different linkage blokes (LDs) or nonrelated traits in the same LD with undesirable effects, resulting in what commonly known as linkage drag [47].

In this study, although these genes still need further investigation, the detected oil synthesis-related genes and their elite haplotypes could be potentially used for oil improvement in soybean breeding. The frequency of excellent haplotypes for high oil content continually increased from wild to cultivated soybean. For example, the frequencies of oil-related excellent haplotypes Hap1 and Hap4 of *Glyma12g33320*, Hap4 of Glyma12g13300 and Hap3 of Glyma12g36600 reached at 82.5 and 7.5%, 88.2, and 95.0% in cultivated soybean, suggested that excellent haplotypes could be further utilized during the improvement of soybean seed oil. However, the selection of target traits may result in negative selection of related traits due to pleiotropic trait correlations or LD drag, therefore, it is necessary to consider other important related traits when selecting target traits or utilizing the elite haplotypes in soybean breeding.

## Materials and methods

### Identification of zf_CCCH protein in soybean

The HMM profile of the zf_CCCH family (PF00642, Zinc finger C-× 8-C-× 5-C-× 3-H type (and similar)), was downloaded from Pfam (http://pfam.xfam.org/), which was used to genome-wide search for the *Gmzf_CCCH* protein in the local protein database of soybean (Wm82.a1.v1.1, download at Soybase, https://www.Soybase.org/), using HMMER3.1 software (HMMER 3.1, http://hmmer.org/). To avoid missing *Gmzf_CCCH* family members, the new HMM profile was constructed by hmm-build using an aligned file of a high-quality protein set (E value < 1 × 10^− 20^) in MUSCLE [48], and was used as the query to search for all the *Gmzf_CCCH* members (E value < 0.001) in soybean. All the obtained protein sequences were submitted to the PFAM databases (http://pfam.xfam.org/) and SMART domain search database (http://smart.embl.de/smart/batch.pl) to confirm the structural integrity of the zf_CCCH domain [49]. Finally, the physical and chemical properties of the *Gmzf_CCCH* protein sequences, including the number of amino acids (NAA), molecular weight (MW), and isoelectric point (theoretical pI), were calculated using the online ExPASy tool (https://web.expasy.org/compute_pi/) [50]. The subcellular localization of *Gmzf_CCCH* proteins was predicted via WoLF PSORT (https://wolfpsort.hgc.jp/) [51]. The non-redundant, confirmed genes encoding proteins with zf_CCCH domains were assigned as the family of *Gmzf_CCCH* genes.

### Conserved sequence and phylogenetic analyses

It is important to analyze new gene families with known established families during phylogenetic analyses. Therefore, to further characterize the *zf_CCCH* genes in soybean and its relationship with other legumes such as *Medicago truncatula, Phaseolus vulgaris and Vigna unguiculata*, the zf_CCCH proteins of *Arabidopsis* and rice were added to establish the phylogenetic relationship following the references of Rameneni, et al. [28] and Upadhyay, et al. [52]. The zf_CCCH protein sequences of the *Medicago truncatula* (50 *zf_CCCHs*)*, Phaseolus vulgaris* (43 *zf_CCCHs*) *and Vigna unguiculata* (49 *zf_CCCHs*) were downloaded from the database of plant genomics resource Phytozome 12 using the tool of Biomart with the Pfam ID:PF00642 (https://phytozome.jgi.doe.gov/biomart). The zf_CCCH protein sequences of *Arabidopsis* (68 *zf_CCCHs*) and rice (67 *zf_CCCHs*) was from Wang, et al. [10]. Multiple alignments of all the conserved zf_CCCH protein sequences of soybean and the three legumes with that of *Arabidopsis* and rice were performed using MUSCLE [48] with default parameters. A phylogenetic tree was constructed using the IQ-TREE software via a maximum-likelihood method with 1000 bootstrap replications [53]. Figtree 1.4.4 (http://tree.bio.ed.ac.uk/software/figtree/) was used to optimize visualization of the phylogenetic tree and classify sequences to groups.

### Chromosomal locations and Synteny analysis

The information of *Gmzf_CCCH* gene loci on soybean chromosomes was extracted according to the annotation gff3 file (Wm82.a1.v1.1) using a Perl script. Multiple Collinearity Scan toolkit (MCScanX) was adopted to analyze the gene Synteny with following parameters (five genes required to call a collinear block based on the previous all-to-all BLASTP result E-value ≤1 × 10^− 5^) [31]. Tandem duplication events were defined as two or more adjacent homologous genes located on the same chromosome without any intervening genes, while segmental duplication genes were defined as those located in the same synteny blocks. The chromosomal distribution, segmental duplication of *Gmzf_CCCH* genes were visualized by the ‘circlize’ package of R program [54]. The ratio of nonsynonymous substitutions per nonsynonymous site (Ka) to synonymous substitutions per synonymous site (Ks) was computed using the function ‘Simple Ka/Ks Calculator’ of TBtools [55]. Then, the mean Ks values (T = Ks/2λ) were used to calculated the estimated date (MYA, million years ago) of each duplication event, assuming clocklike rates (λ) of 6.1 × 10^− 9^ [56].

### Characterization of gene structure, protein domain, and motif

The domains of 116 members of *Gmzf_CCCH* gene family in soybean were confirmed by SMART domain search database (http://smart.embl.de/smart/batch.pl) [49]. The conserved motifs of the *Gmzf_CCCH* gene family in soybean were determined by the online MEME suite program (http://meme-suite.org) [57]. The gene structure, protein domain, and motif of the *Gmzf_CCCH* genes were visualized using the ‘Gene Structure View’ function of TBtools [55] according to the annotation gff3 files of Wm82.a1.v1.1, and the protein domain file of SMART domain search as well as the Motif result files of MEME suite.

### Identification of putative cis-acting regulatory elements

The 2 kb upstream sequences of the *Gmzf_CCCH* genes were extracted from the soybean genome reference (Wm82.a1.v1.1) using the ‘GTF/GFF3 Sequences Extract’ function of TBtools [55], and submitted to PlantCARE (http://bioinformatics.psb.ugent.be/webtools/plantcare/html/) for the prediction of potential cis-acting elements.

### Expression profiles of Gmzf_CCCH genes

To analyze the expression profiles of *Gmzf_CCCH* genes in different tissues, the public RNA-seq data, including data files of Severin, et al. [36], GSE42871 [38], E-MTAB-4270 [37], were obtained from Soybase (https://www.Soybase.org/), National Center for Biotechnology Information Gene Expression Omnibus (https://www.ncbi.nlm.nih.gov/geo/query/acc.cgi) and Expression Atlas of EMBL-EBI (https://www.ebi.ac.uk/gxa/experiments), then visualized using the ‘pheatmap’ package of R software [58].

### Selective regions and QTLs around GmZf_CCCH genes

According to the results of soybean domestication and improvement regions in previous reports [39–42], we identified whether the *Gmzf_CCCH* genes were in the domestication or improvement regions or not based on physical position. The results of QTLs and GWAS in soybean were downloaded from Soybase (https://www.Soybase.org/). According to Wm82.a1.v1.1 genome annotation, the QTLs around *Gmzf_CCCH* genes (upstream and downstream 100 kb) were anchored on the corresponding physical position of genome. The distribution of *Gmzf_CCCH* genes, domestication and improvement regions and related QTLs were visualized by the ‘circlize’ package of R program [54].

SNP (Single nucleotide polymorphism) data gathered from resequencing of 302 soybeans (including *Glycine soja* and *Glycine max* (landrace and improved cultivar)), downloaded under SRA: SRP045129 of NCBI dbSNP, were used to detected the genetic variation of *Gmzf_CCCH* genes in 302 soybeans [39]. The SNPs of *Gmzf_CCCH* genes (including upstream promoter 2 kb and gene sequence) in 302 soybeans with missing data > 10% or MAF (Minor Allele Frequency) < 5% were filtered. In addition, the lines in each panel for more than 25% missing data were also filtered. Marker missing data was imputed using the LD-kNNi genotype imputation method [59] in TASSEL 5.25 [60]. The SNP data of *Gmzf_CCCH* genes in 302 soybeans were further used to analyze the genetic diversity and construct the phylogenetic tree using IQ-TREE software [53].

### GWAS between Gmzf_CCCH genes and height, seeds weight, protein and oil related traits in soybean

In order to verify the relationship between *Gmzf_CCCH* genes and nearby QTL, and to reveal the functional phenotypes that these genes may affect, a GWAS between 9 traits (including height, seeds weight, protein, oil contain and five fatty acids) of the 164 soybeans (from 302 soybeans) download from GRIN (https://npgsweb.ars-grin.gov/) and the corresponding SNPs data of *Gmzf_CCCH* genes from the resequencing data of 302 soybeans were performed using mix line model of Tassel 5.25 [60]. The Q matrix were calculated by Structure 2.3.4 [61] and the kinship were calculated by default method of Tassel 5.25 [60]. The threshold of significant associations was set as LOD ≥ 2.5 (−log(p) ≥2.5) following the references [62–64]. The *Gmzf_CCCH* genes and their upstream 2 Kb sequence in the released 30 soybean genomes (26 released by Liu, et al. [44] and 4 from soybase, including wild and cultivated soybeans) were extracted for variation analysis of gene sequence.

### Hap analysis of Gmzf_CCCH genes in soybeans

The haplotype analysis of the *Gmzf_CCCH* genes among the 164 of 302 soybean accessions were performed using the SNP data on *Gmzf_CCCH* genes and the promoter sequences by ‘CandiHap’ package [65] of R, and the difference of phenotype corresponding to different haplotypes of genes were tested with TukeyHSD method [66]. The frequency of haplotype among wild, landraces and cultivar soybean were also calculated. The results were visualized using ‘ggplot2’ [67] and ‘ggpubr’ [68] packages of R.

## Conclusions

We identified and characterized the zf_CCCH gene family in soybean. A total of 116 *Gmzf_CCCH*s were obtained and classified into subfamilies 1–13 after systematic investigations. Gene duplication and expansion analysis showed that tandem and segmental duplications contributed to the expansion of the *Gmzf_CCCH* gene family, and segmental duplication play the main role. Purifying selection was the major driving force in *Gmzf_CCCH* gene family evolution. The analyses of conserved domains and motifs suggested that, in general, adjacent members collinear (or duplicated) gene pairs in the phylogenetic tree had common motif compositions. The *Gmzf_CCCH*s were involved in regulating various biological processes, such as plant growth and development, phytohormone-mediated metabolism, and defensive responses to various abiotic and biotic stresses. The expression patterns of *Gmzf_CCCH* genes were tissue-specific, the highly similar genes in sequence, especially collinear (or duplicated) gene pairs in one cluster clad exhibited similar expression patterns. The GWAS and haplotype analysis for *Gmzf_CCCH* genes in the 164 soybeans of 302 resequencing accessions in Zhou, et al. [39], certificated 5 genes and newly found six genes in the domesticated region. And in addition, 11 genes were detected as domestication genes involving in the regulation of oil and protein synthesis and metabolism or plant growth in soybean. This study provides a scientific foundation for the comprehensive understanding, future cloning and functional studies of zf_CCCH genes in soybean, and provides a systematic and effective method for identifying family genes, predicting and studying the function of family gene members, meanwhile, it was also be helpful for the improvement of soybean with high oil content using the *Gmzf_CCCH* genes.

## Supplementary Information

**Additional file 1.**

**Additional file 2.**

## Data Availability

The soybean reference genome assembly (Wm82.a1.v1.1) and its GFF3 gene annotation, as well as the *Gmzf_CCCH* coding sequences and protein sequences, are available Soybase website (https://www.Soybase.org/). The released 30 soybean genomes (26 released by Liu, et al. [44]) are available Soybase website (https://www.Soybase.org/). The HMM profile of the *zf_CCCH* family (PF00642, Zinc finger C-× 8-C-× 5-C-× 3-H type (and similar)), was downloaded from Pfam database (http://pfam.xfam.org/ family/PF00642). The *zf_CCCH* proteins of *Arabidopsis* and rice were download from the database of The Arabidopsis Information Resource (TAIR, https://www.arabidopsis.org/index.jsp) and Rice Genome Annotation Project website (http://rice.plantbiology.msu.edu/), respectively, according to the reference of Wang, et al. [10]. The expression profiles of *Gmzf_CCCH* genes in different tissues at different developmental stages are available on Soybase (https://www.Soybase.org/) [36], National Center for Biotechnology Information Gene Expression Omnibus under the accession number of GSE42871 (https://www.ncbi.nlm.nih.gov/geo/query/acc.cgi?acc=GSE42871) [38], and the Expression Atlas of EMBL-EBI under the accession number of E-MTAB-4270 (https://www.ebi.ac.uk/gxa/experiments/E-MTAB-4270) [37], respectively. The public results of QTLs and GWAS in soybean were downloaded from Soybase (https://www.Soybase.org/). The SNP (Single nucleotide polymorphism) data for *Gmzf_CCCH* genes gathered from resequencing of 302 soybeans was downloaded from Figshare database (http://figshare.com/articles/Soybean_resequencing_project/1176133) [39]. The phenotype data (including height, seeds weight, protein, oil contain and five fatty acids) of the 164 soybeans (from 302 soybeans) download from GRIN (https://npgsweb.ars-grin.gov/) according to the accession ID of Table S11.

## Abbreviations

zf_CCCH: CCCH zinc finger
Gmzf_CCCH: *Glycine max* CCCH zinc finger
GWAS: Genome-Wide Association Studies
MCScanX: Multiple Collinearity Scan toolkit
LOD: Logarithm of Odds
QTL: Quantitative Trait Loci
WGD: Whole genome duplication
MeJA: Methyl Jasmonate
SA: Salicylic Acid
ABA: Abscisic Acid
GA: Gibberellins
IAA: Auxin
MW: Molecular Weight
pI: Protein Isoelectric Point
NAA: Amino Acids
MAF: Minor Allele Frequency
SNP: Single Nucleotide Polymorphism
