# Supplementary material for: The CCCH zinc finger family of soybean (Glycine max L.): genome-wide identification, expression, domestication, GWAS and haplotype analysis
Source: BMC Genomics. 2021 Jul 7;22:511. doi: 10.1186/s12864-021-07787-9 (PMC8261996; doi:10.1186/s12864-021-07787-9)
Supplement: Supplementary file 2 — Additional file 2. [file 12864_2021_7787_MOESM2_ESM.docx]

**
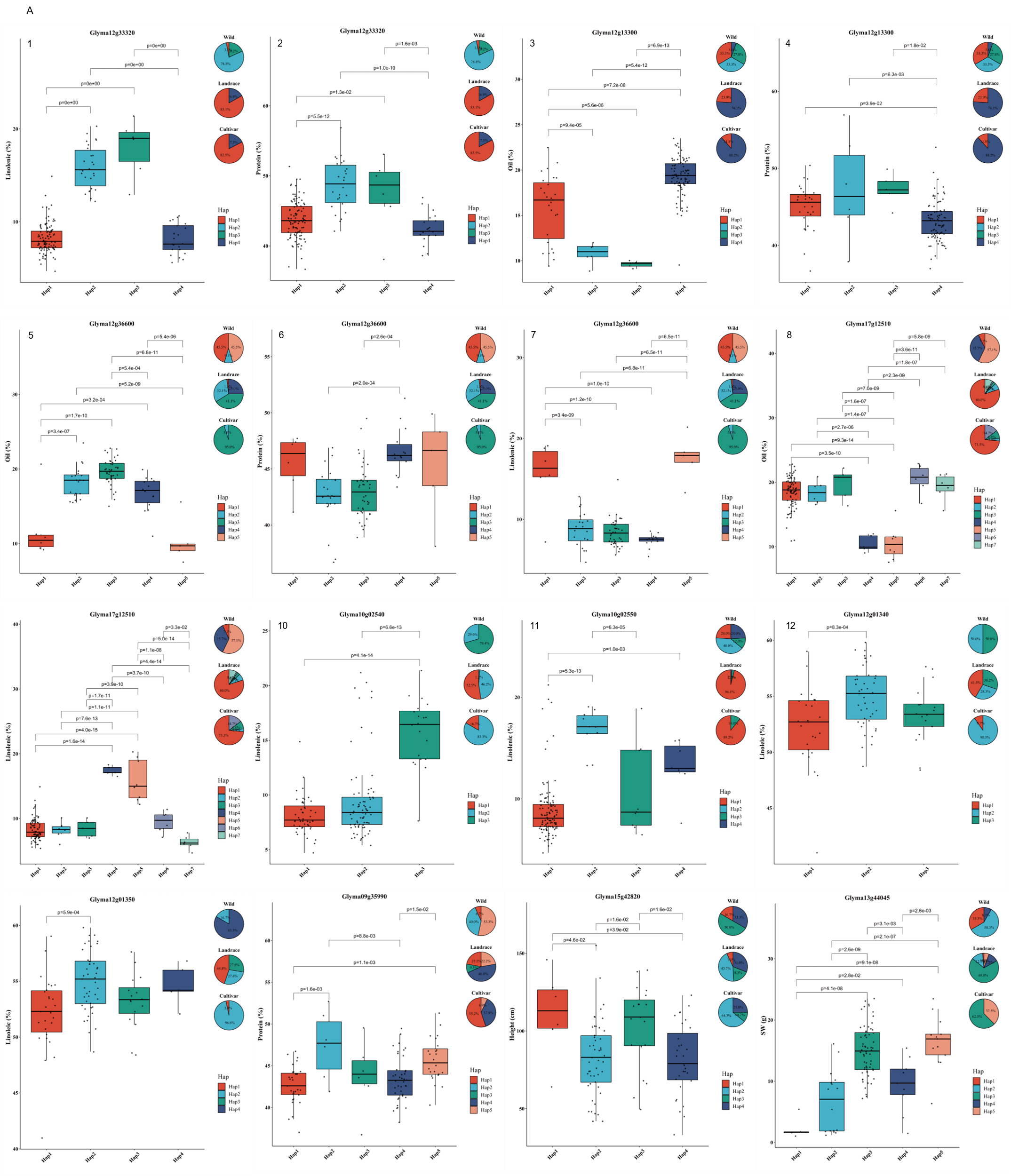
**

**Figure S1 A**

**
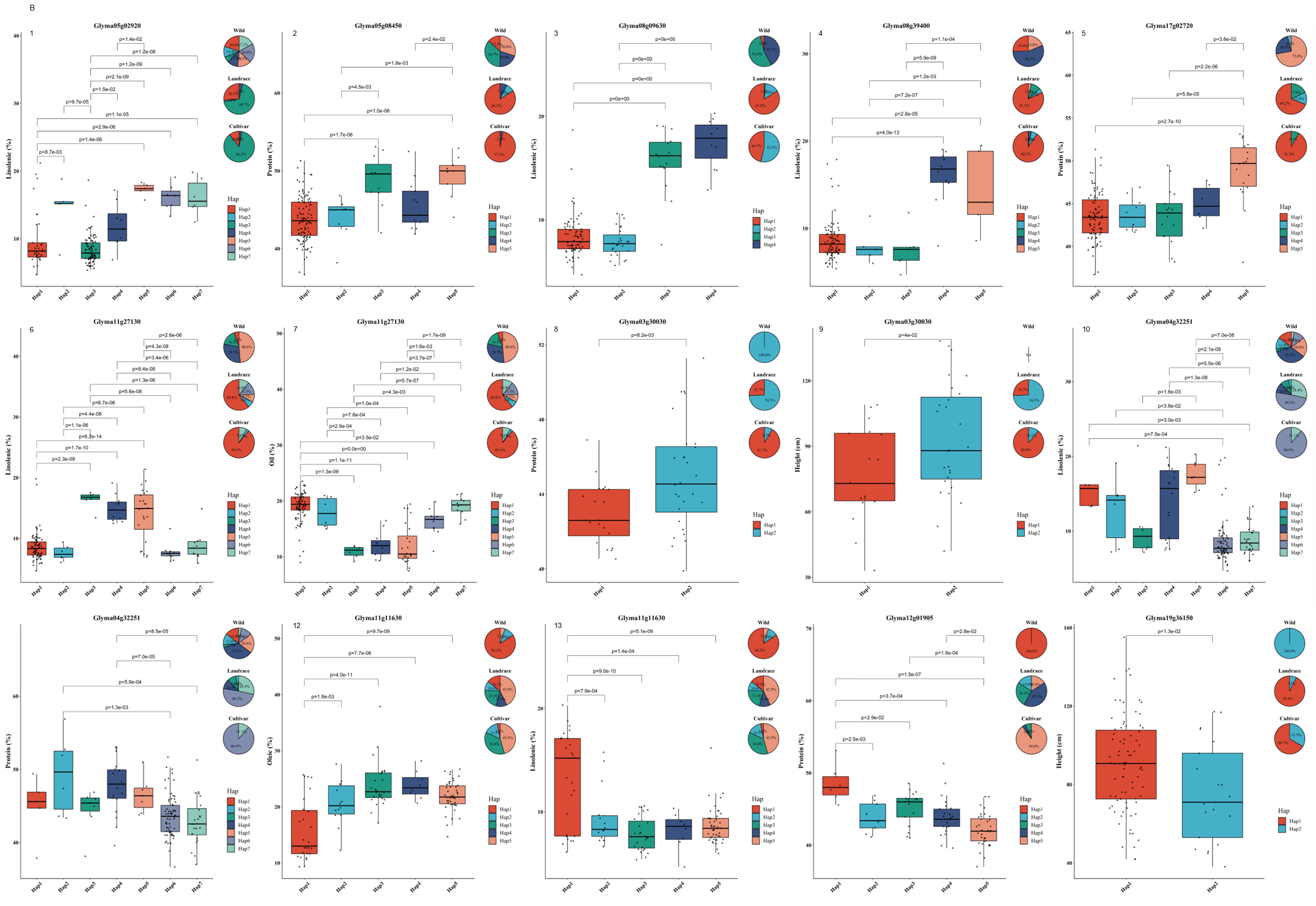
**

**Figure S1 B**

**Figure S1**: The haplotype, the frequency and the corresponding phenotype of haplotype for 22 *Gmzf_CCCH*s in 164 of 302 soybean accessions. (**A**)**,** haplotype analysis for 11 *Gmzf_CCCH*s domestication gene; (**B**)**,** haplotype analysis for 11 *Gmz_CCCH* genes in domestication regions.
